# Dynamic bacterial community response to *Akashiwo sanguinea* (Dinophyceae) bloom in indoor marine microcosms

**DOI:** 10.1038/s41598-021-86590-8

**Published:** 2021-03-26

**Authors:** Seung Won Jung, Junsu Kang, Joon Sang Park, Hyoung Min Joo, Sung-Suk Suh, Donhyug Kang, Taek-Kyun Lee, Hyun-Jung Kim

**Affiliations:** 1 Library of Marine Samples, Korea Institute of Ocean Science and Technology, Geoje, 53201 Republic of Korea; 2 Department of Oceanography, Pukyoung National University, Busan, 48513 Republic of Korea; 3 Division of Polar Ocean Science, Korea Polar Research Institute, Incheon, 21990 Republic of Korea; 4 Department of Bioscience, Mokpo National University, Muan, 58554 Republic of Korea; 5 Maritime Security Research Center, Korea Institute of Ocean Science and Technology, Busan, 49111 Republic of Korea; 6 Risk Assessment Research Center, Korea Institute of Ocean Science and Technology, Geoje, 53201 Republic of Korea

**Keywords:** Ecology, Ocean sciences

## Abstract

We investigated the dynamics of the bacterial composition and metabolic function within *Akashiwo sanguinea* bloom using a 100-L indoor microcosm and metagenomic next-generation sequencing. We found that the bacterial community was classified into three groups at 54% similarity. Group I was associated with “during the *A. sanguinea* bloom stage” and mainly consisted of Alphaproteobacteria, Flavobacteriia and Gammaproteobacteria. Meanwhile, groups II and III were associated with the “late bloom/decline stage to post-bloom stage” with decreased Flavobacteriia and Gammaproteobacteria in these stages. Upon the termination of the *A. sanguinea* bloom, the concentrations of inorganic nutrients (particularly PO_4_^3−^, NH_4_^+^ and dissolved organic carbon) increased rapidly and then decreased. From the network analysis, we found that the *A. sanguinea* node is associated with certain bacteria. After the bloom, the specific increases in NH_4_^+^ and PO_4_^3−^ nodes are associated with other bacterial taxa. The changes in the functional groups of the bacterial community from chemoheterotrophy to nitrogen association metabolisms were consistent with the environmental impacts during and after *A. sanguinea* bloom. Consequently, certain bacterial communities and the environments dynamically changed during and after harmful algal blooms and a rapid turnover within the bacterial community and their function can respond to ecological interactions.

## Introduction

The marine bacterial community is a fundamental component contributing to decomposition and primary production within marine ecosystems and is a major driver of nutrient cycling in coastal waters^1^. The interactions between phytoplankton and the bacterial community play important roles in shaping the environment by influencing the biogeochemical cycles^2^. In particular, the phycosphere (the environment around the phytoplankton cells where bacteria feed on the carbohydrate sources released by the phytoplankton^2^) and the phytoplankton rely on the bacteria to remineralise organic matter into inorganic substrates that enhance growth^3^. Therefore, the relationships between bacteria and phytoplankton communities, particularly harmful algal blooms (HABs), are a promising research field in bacterial ecology because they provide insights into the functional traits of individual populations and how bacterial communities are controlled by HAB growths, as well as how these communities are interlinked^4^.

Diverse marine microbial communities interacted with other marine organisms; specifically, they have the potential to affect HABs^5^. Importantly, interactions between HABs and bacteria shape their environment^6^. Yang et al.^7^ described how abundances of heterotrophic bacteria rapidly increased after *A. sanguinea* HABs and DO concentration dropped as a result of bacterial decomposition. Recently, specific bacterial phylotypes have been identified as associated with different microalgae. Yang et al.^8^ reported species-specific relationships between bacterial communities and *A. sanguinea* bloom. Our collaborated study^9^ discussed that certain bacterial communities were closely related to *A. sanguinea* bloom. In particular, *Polaribacter marinivivus* may have a symbiotic association with *A. sanguinea* bloom, while *Marinovum algicola* may be inhibited in HABs. Therefore, it is important to elucidate the ecological role of specific bacteria associated with HABs.

The environmental changes and their interactions with bacteria and HABs can increase our understanding of the microbial ecology. With the advancement of metagenomic next-generation sequencing (mNGS), a big data (large volume of sequencing) has been analysed. Recently, molecular ecological studies have used mNGS to estimate changes in population and community dynamics^10^. In addition, new technologies for studying aquatic microbial diversity require smaller volumes and nanograms of DNA^11^. Microcosm or mesocosm experiments mimic real ecosystems^12,13^, and can clarify the effects of changes in biotic and abiotic factors, providing an important link between laboratory and field data^14,15^. Our previous research revealed how bacterial composition changed with *A. sanguinea* blooms, similar to natural ecosystem environments^9^. In this study, we explored the genetic dynamics of the bacterial community during *A. sanguinea* bloom and after its termination using indoor 100-L microcosms. We also investigated bacterial community distributions and their functions as major decomposers in microcosms following the termination of an *A. sanguinea* bloom.

## Results

### Changes in environmental parameters correlated with *A. sanguinea* abundance

At the initiation of the experiment, *A. sanguinea* cells bloomed with a mean abundance of 920 cells mL^-1^ at the average water temperature of 18 °C. After decreasing the water temperature to 16 °C on day 4, the abundance rapidly decreased, and *A. sanguinea* cells disappeared on day 9 (Supplementary Fig. S1). The pH, dissolved oxygen and nitrate concentrations decreased similarly with the number of *A. sanguinea* cells. After the rapid decrease in *A. sanguinea* abundance, the NH_4_^+^ concentration increased rapidly for 7 days, peaked at 204 ± 12 μM on day 14, and then gradually decreased. A similar pattern occurred with PO_4_^3−^. Conversely, the SiO_2_^-^ concentrations remained between 7.85 and 8.39 μM and did not vary significantly throughout the experimental period. The dissolved organic carbon (DOC) concentration decreased with decreasing *A. sanguinea* abundance and then slightly increased with the termination of the HABs. The chlorophyll *a* concentration showed similar changes with the *A. sanguinea* abundance and significantly correlated with changes in the HABs.

### Microbial succession patterns during and after *A. sanguinea* bloom

**Figure 1:**
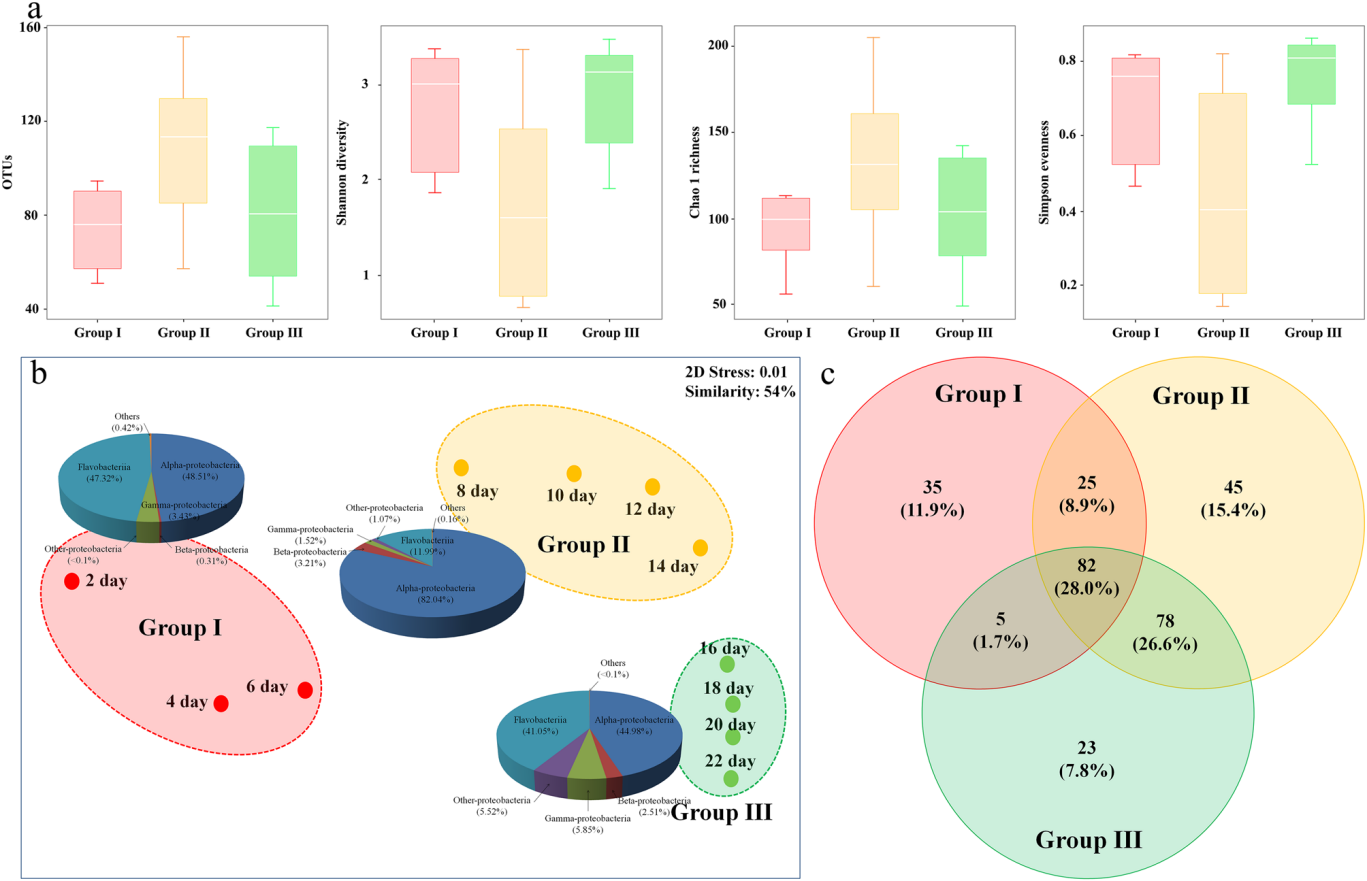
Alpha diversity and the distribution of operational taxonomic units (OTUs) in the bacterial community during *Akashiwo sanguinea* bloom (Group I) and late/decline stage (Group II) to post-bloom stage (Group III)” within an indoor microcosm. (**a**) Box plots of alpha diversity (median, min and max) showing the numbers of OTUs, Shannon diversity, Chao1 richness and Simpson evenness. (**b**) Non-metric multidimensional scaling plot using the Bray–Curtis similarity method. The pie charts indicate the high-ranking taxonomic distribution at the class level of the bacterial community. (**c**) Venn diagram showing the shared and unique bacterial operational taxonomic units. Figure 1c was generated with Veeny v2.1.0 (https://bioinfogp.cnb.csic.es/tools/venny).

In the indoor microcosm, the total bacteria abundance increased rapidly for the first 7 days, decreased, increased again, and remained constant until the end of the experiment (Supplementary Fig. S1). The number of operational taxonomic units (OTUs) and alpha diversity showed similar trends similar to the read counts (Fig. 1a). The bacterial community was classified into three groups at 54% similarity using non-metric multidimensional scaling (nMDS) analysis (Fig. 1b). Group I was associated with “during *A. sanguinea* bloom stage” comprising communities of Alphaproteobacteria (48.51%), Flavobacteriia (47.32%), and Gammaproteobacteria (3.43%). Groups II and III were associated with “late bloom / decline stage to post-bloom stage”, respectively. Flavobacteriia and Gammaproteobacteria decreased in Group II but increased again to 41.05% and 5.85% in Group III, respectively. The resulting Venn diagrams showed that bacterial species in Groups I, II, and III represented 147, 234 and 188 taxa, respectively. In all groups, the common bacteria overlapped in 82 taxa (28.0% of the total number of bacteria) (Fig. 1c). Groups II and III had the largest OTU overlap of 160 taxa, whereas Groups I and III had the smallest OTU overlap of 87 taxa.

**Figure 2:**
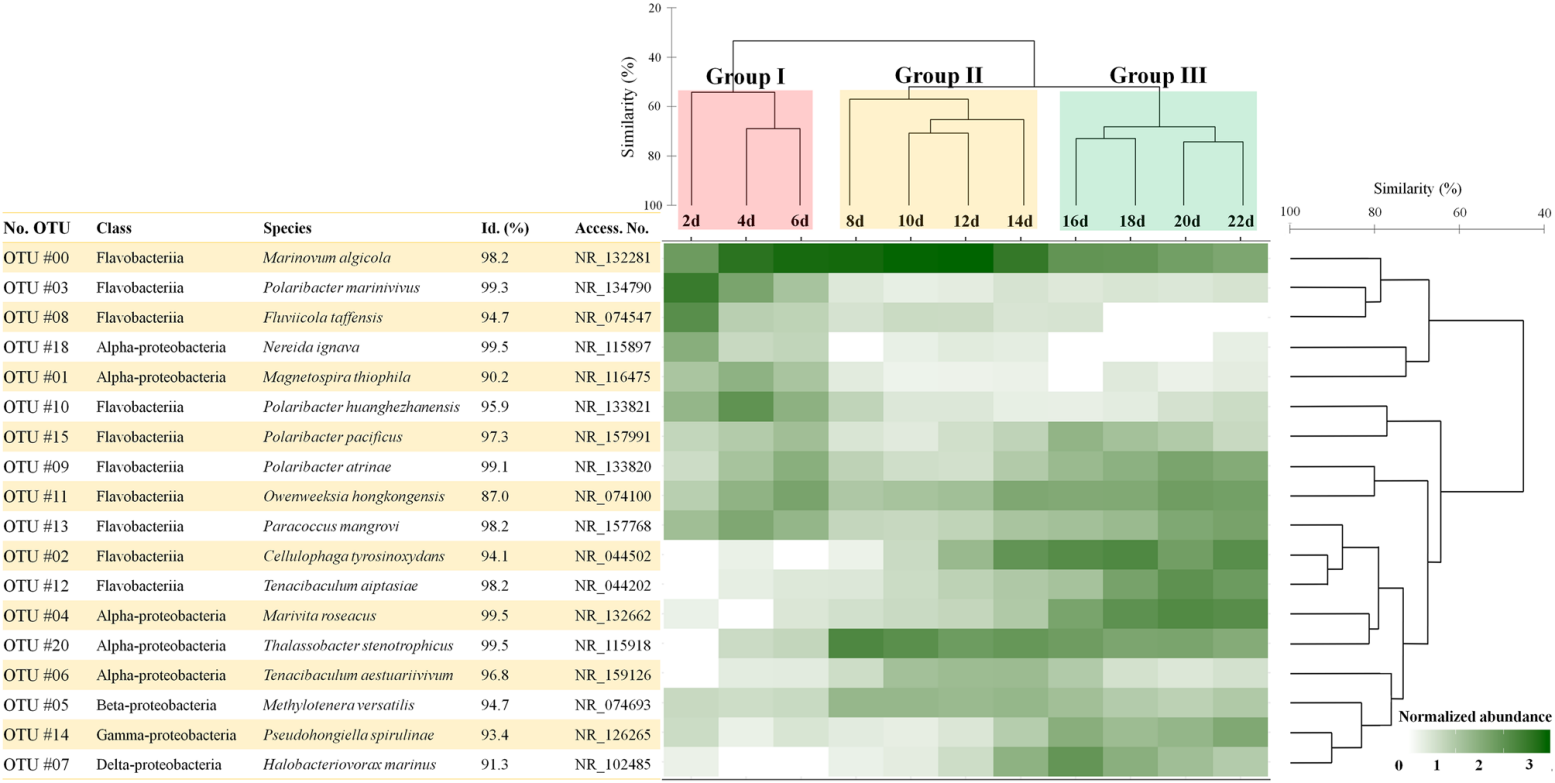
Heatmap showing the most abundant bacterial operational taxonomic units (OTUs). Each OTU displayed a relative abundance of > 1% in at least one sample. The heatmap displayed the fourth root-normalized data at the range of 0.0 to 3.0. Hierarchical agglomerative clustering using the group average of the most abundant bacterial OTUs using the Bray–Curtis similarity method. This figure was generated with RStudio v.1.2.5042 (https://rstudio.com).

The common bacterial species in each group displaying a relative abundance of > 1% in at least one sample included 18 taxa. The accumulated relative abundance of these 18 common bacteria had a mean of 92.50% (Fig. 2). “During *A. sanguinea* bloom stage” (Group I), 10 bacterial OTUs were common taxa, and OTUs # 00 (*Marinovum algicola*), #03 (*Polaribacter marinivivus*), #08 (*Fluviicola taffensis*) and #10 (*Polaribacter huanghezhanensis*) in Flavobacteriia were dominant with the mean relative abundances of 39.72%, 15.55%, 6.82% and 7.87%, respectively. Particularly, OTUs #00, #03 and #08 were clustered in one group with 79% similarity. In Group II (“late bloom/decline stage”), nine bacterial OTUs were common taxa. In particular, OTUs # 00 and #01 (*Magnetospira thiophila*, Alphaproteobacteria) rapidly increased with 61.13% and 17.17%, respectively. In Group III (“post-bloom stage”), 12 bacterial OTUs were common taxa. Interestingly, OTU # 00 (the most dominant OTU in Groups I and II) decreased in Group III to 11.81%. The following OTUs newly and/or rapidly increased: #02 (*Cellulophaga tyrosinoxydans*, 18.53%), #13 (*Paracoccus mangrove*, 9.80%), and #09 (*Polaribacter atrinae*, 5.19%) in Flavobacteriia; #14 (*Pseudohongiella spirulinae*, 4.25%) in Gammaproteobacteria; # 04 (*Marivita roseacus*, 16.92%) and #06 (*Tenacibaculum aestuariivivum*, 8.26%) in Alphaproteobacteria; and #07 (*Halobacteriovorax marinus*, 5.51%) in Deltaproteobacteria. The Venn diagram indicated that bacterial species in Group I, II and III represented 147, 230 and 188 taxa, respectively. In all samples, the common bacteria overlapped in 82 taxa (28.0% of the total number of bacteria) (Fig. 1c). In particular, during the *A. sanguinea* bloom stage (Group I), the numbers of bacterial OTUs consisted of only 35 taxa. Although these taxa were rare at < 1%, four taxa of OTUs including #57 (*Fluviicola hefeinensis*), #74 (*Bizionia arctica*) and #48 (*Tenacibaculum jejuense*) in Flavobacteriia and #43 (*Litoricola marina*) in Gammaproteobacteria were detected at a relative abundance of 1–0.1%.

### Impact of common bacteria in relation to environmental factors

**Figure 3:**
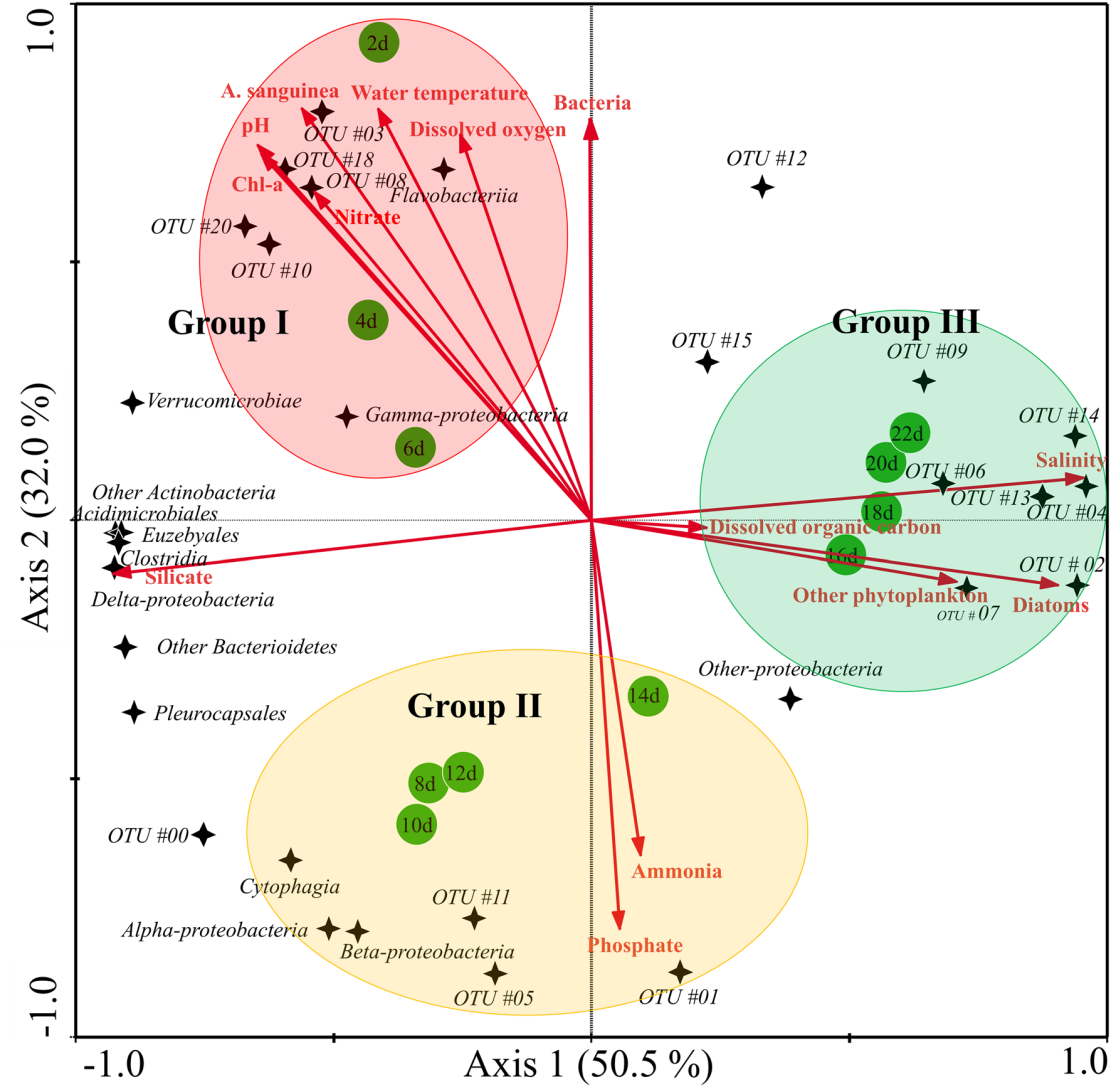
Redundancy analysis (RDA) ordinations of the most abundant bacterial operational taxonomic units (OTUs) associated with environmental and phytoplankton factors. Each OTU displays a relative abundance of > 1% in at least one sample. The lengths and angles of the arrows represent the correlations between the environmental and phytoplankton factors (red lines) and the first two RDA axes. The sampling day and the common bacterial OTUs (at the class level) are indicated green circles and black stars, respectively. Groups I − III are obtained from the nMDS analysis presented in Fig. 1b. The identified bacterial species with OTU numbers are displayed in the heatmap in Fig. 2. This figure was generated with CANOCO program v4.55 (http://www.canoco5.com).

On the Redundancy analysis (RDA) biplot in Fig. 3, the lengths of the arrows indicate the relative importance of the variables in explaining the data set of environments and phytoplankton communities. The angle of the arrows relative to the axes and to each other indicate the strength of their correlation (Fig. 3). The common bacteria–environment relationship represented in the plot includes 14 variables that accounted for 50.5% (first axis) and 32.0% (second axis). In Group I, the cluster in the upper-left quadrant of OTUs #3, #8, #10, #18 (*Nereida ignava*) and #20 (*Thalassobacter stenotrophicus*) were strongly associated with the arrows of *A. sanguinea*, chlorophyll *a*, pH, water temperature, dissolved oxygen and nitrate factors. This cluster reflects the influence of the *A. sanguinea* bloom. In Group II, the cluster in the lower-left quadrant the OTUs #00, #01, #05 (*Methylotenera versatilis*) and #11 (*Owenweeksia hongkongensis*) were associated with the PO_4_^3−^ and NH_4_^+^ arrows. This cluster reflects their rapid increases with decreasing *A. sanguinea* bloom. In the Group III cluster in upper-right quadrant the OTUs #02, #04, #06, #07, #09, #13 and #14 were associated with DOC, diatoms (common species, *Pseudo-nitzschia delicatissima,* and *Cylindrotheca closterium*) and other phytoplankton (autotrophic nanoflagellates) arrows. The Group three cluster reflects the succession of other phytoplankton communities after the termination of the *A. sanguinea* bloom.

**Figure 4:**
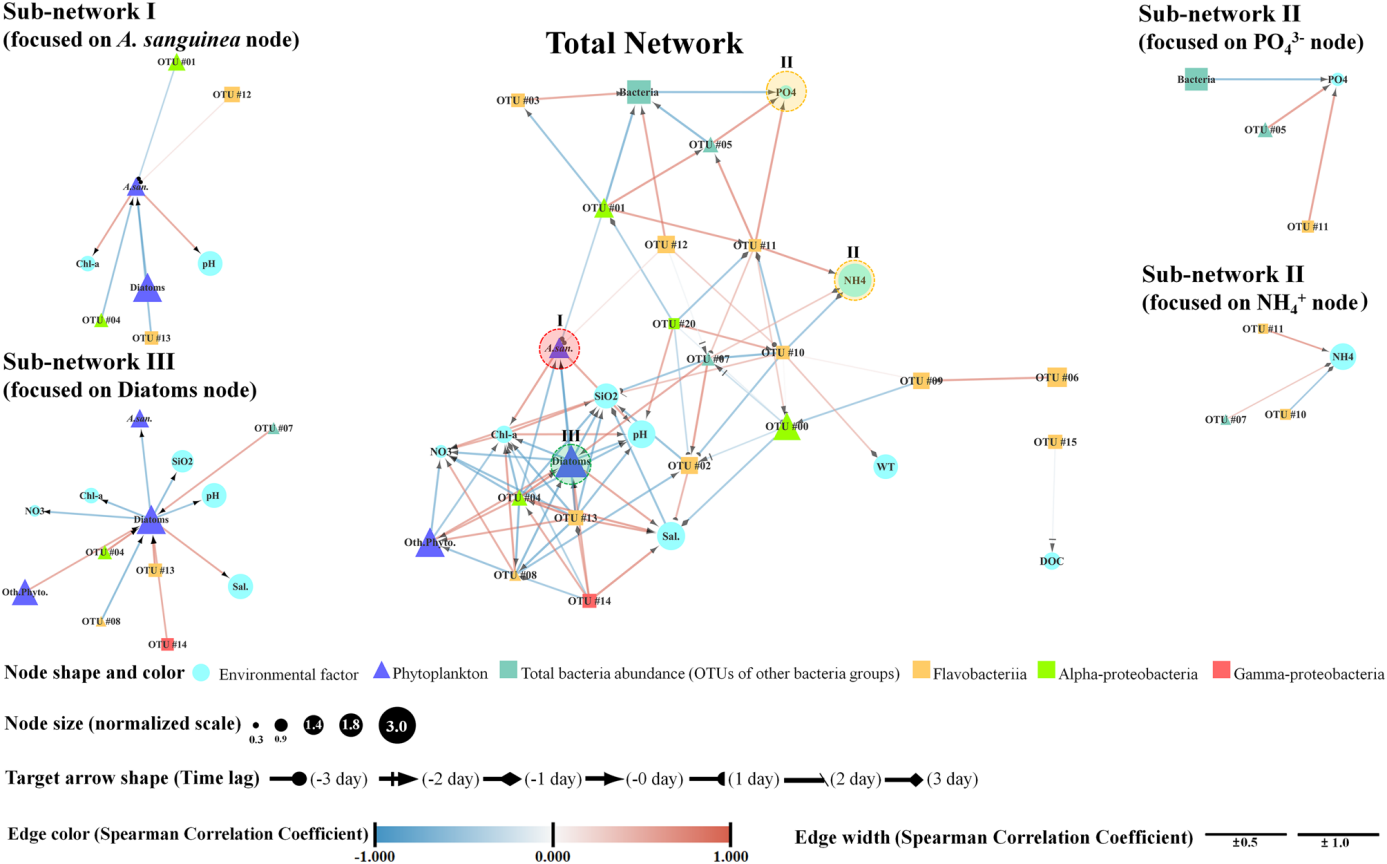
Network analysis derived from the most abundant bacterial operational taxonomic units (OTUs) and the environmental and phytoplankton factors with statistically significant correlations with bacterial OTUs (*P* < 0.01; false discovery *Q* < 0.05). Zoomed images are the sub-networks (I-III) are associated with *A. sanguinea*, NH_4_^+^, PO_4_^3−^, diatom nodes and selected adjacent edges. The identified bacterial species with OTU numbers are displayed in the heatmap on Fig. 2. This figure was generated with Cytoscape v3.7.2 (https://cytoscape.org).

The network analyses involving the common bacterial OTUs, environmental factors, and *A. sanguinea* bloom exhibited distinct interactions with specific bacteria communities and environmental factors (Fig. 4, Supplementary Tables S1 and S2). The network significantly correlated bacterial species and environmental factors with 25 nodes and 90 edges. Environmental factors, including pH and chlorophyll *a*, were significantly associated with *A. sanguinea* bloom, and the HABs had strong negative associations with OTUs #13 (SCC = –0.86, delay time = 0 day) and #04 (–0.85, 0 day). The abundance of the diatom communities negatively correlated with chlorophyll *a* and the *A. sanguinea* bloom. OTUs #07 (0.81, 0 day), #04 (0.98, 0 day), #13 (0.97, 0 day) and #14 (0.80, 0 day) possessed strong positive correlations with diatom abundance. Conversely, #08 was negatively correlated (–0.94, 0 day) with diatom abundance. NH_4_^+^ showed strong positive correlations with OTU #11 (0.88, 0 day), whereas it was negatively correlated with OTU #10 (–0.77, –1 day). PO_4_^3−^ had strong positive correlations with OTUs #11 (0.91, 0 day) and #05 (0.91, 0 day).

### Functional prediction for presented bacterial community

**Figure 5:**
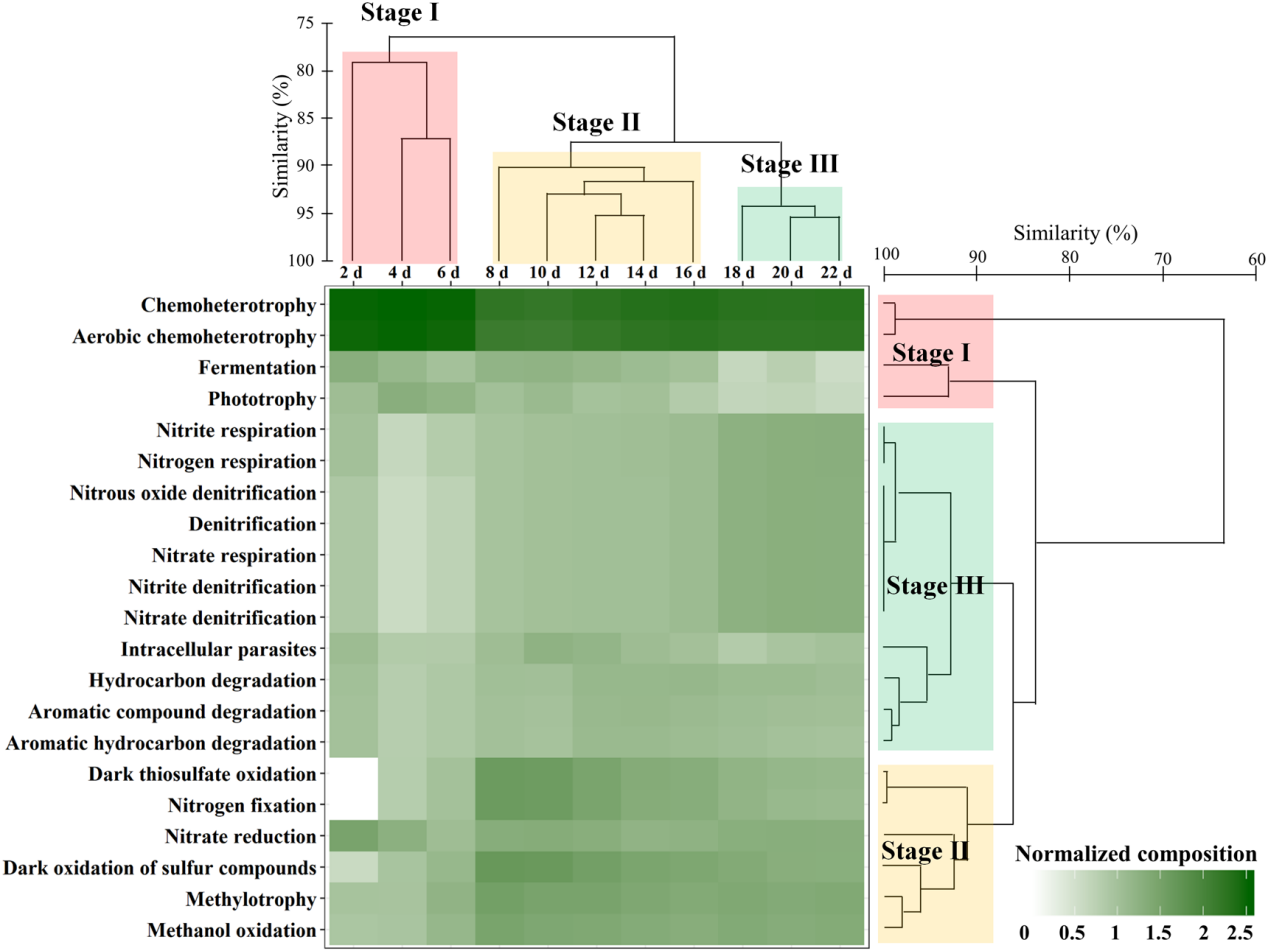
The time-series heatmap showing the metabolic functions during the changes in bacterial community composition using functional annotation analysis of the prokaryotic taxa (FAPROTAX). The heatmap displays the fourth root-normalized data of the bacterial read counts. The time series and functional groups of the bacterial operational taxonomic units are clustered using the Bray–Curtis similarity method. This figure was generated with RStudio v.1.2.5042 (https://rstudio.com).

A total 494 bacterial OTUs were identified using the pipeline of functional annotation of prokaryotic taxa (FAPROTAX) to evaluate the potential functional differences among stages (Fig. 5). The functional profiles of stage I showed increases in chemoheterotrophy, aerobic chemoheterotrophy, methylotrophy, methanol oxidation, nitrate reduction, fermentation, phototrophy, photoheterotrophy and aerobic anoxygenic phototrophy. In particular, the functions of chemoheterotrophy and aerobic chemoheterotrophy were the most abundant, averaging 30.51% and 27.86% of the total functional groups, respectively. In stages II and III, the major functional group chemoheterotrophy and aerobic chemoheterotrophy decreased relatively. The phytotrophy, photoheterotrophy and aerobic anoxygenic phototrophy also decreased. Conversely, functional groups associated with the transformation of sulphur, nitrogen and hydrocarbon sources (i.e., dark oxidation of sulphur compounds, dark thiosulfate oxidation, nitrogen fixation, nitrogen respiration, nitrite denitrification, hydrocarbon degradation and aromatic hydrocarbon degradation) increased in abundance when compared to stage I.

## Discussion

HABs are a common global ecological issue in coastal waters^7,16^. In this study, we demonstrated that environmental parameters change with the destruction of *A. sanguinea* in a closed artificial ecosystem, increasing bacterial abundances. PO_4_^3−^ and NH_4_^+^ concentrations strongly increased with decreased *A. sanguinea* abundance. In our previous mesocosm study, we identified strong correlations between phosphorus and nitrogen sources, and inorganic nutrient concentrations increased significantly after the lysis of HABs^14^. Thus, the increase in specific inorganic nutrients is the result of the degradation of the released substrates from the destruction of *A. sanguinea* cells by specific bacteria. Field monitoring research^9^ demonstrated that *A. sanguinea* blooms rapidly decreased when water temperature was below 16 °C (Fig. 1). Our microcosm experiment results proved that *A. sanguinea* growth was inhibited at water temperature below approximately 15 °C. Du et al.^17^ reported the growth of a *A. sanguinea* bloom was inhibited in temperatures below 12 °C. Lee et al.^18^ suggested HABs are inhibited in water temperatures below 16 °C in the neighbouring Southern sea of Korea. Previous research has suggested the optimum growth temperature for *A. sanguinea* is quite diverse. For example, in the Xiamen Sea of East China, *A. sanguinea* grew in a wide temperature range of 18–30 °C^19^. In the laboratory, the growth temperature of HABs were relatively higher (over 20 °C) than those obtained in field studies^20,21^. Therefore, both microcosm studies and our previous field results^8^ suggest temperature may be a limiting factor for the growth of *A. sanguinea* blooms. It appears variations in population growth depend on the regional habitats and temperatures below 16 °C limits growth in the Southern sea of Korea.

Rapid changes in the structure of the bacterial community reflected the changes in the environmental conditions as demonstrated by the distinct response stages of the *A. sanguinea* bloom. We identified two stages, namely “during *A. sanguinea* bloom stage” (Group I) and “late bloom / decline stage to post-bloom stage” (Groups II and III). During the *A. sanguinea* bloom (Group I), Alphaproteobacteria (44.00%) and Flavobacteriia (41.68%) were the dominant bacterial groups. Other natural or mesocosm phytoplankton studies have revealed specific bacterial taxa (e.g., *Roseobacter* in Alphaproteobacteria and *Flavobacterium* in Flavobacteriia) are commonly associated with the blooms^22,23^. Their ecological functions include nutrient provision, release of organic compounds and competition with algae for a specific ecological niche^24–27^. Furthermore, many researchers believe that bacterial community structure affects phytoplankton through mutualistic and parasitic interactions that regulate the initiation, duration and disappearance of HABs^28–32^.

In this stage (Group I), *Marinovum algicola* (OTU #00, Flavobacteriia) is the pre-dominant taxon. The trend in changes in OTU #00 was opposite to *A. sanguinea* cells, with dramatic decreases in OTU #00 levels after the *A. sanguinea* bloom terminated. This indicates that the bacterium has algicidal activity or inhibitory effects on the host *A. sanguinea*. Further, in this stage, the investigated environmental factors (PO_4_^3−^, NO^3−^, DOC, pH and chlorophyll *a*) all rapidly decreased, whereas the total bacterial abundance rapidly increased. These rapid decreases in PO_4_^3−^, NO^3−^ and DOC concentrations may be due to the increased uptake as energy sources by the bacteria^33^. Our findings showed similar changing patterns as Gao et al.^34^ and Jung et al.^13^, in which the Alphaproteobacterial abundance negatively correlated with NH_4_^+^, PO_4_^3−^ and DOC concentrations. The change in the relative abundances of OTUs #03 (*Polaribacter marinivivus*) and #08 (*Fluviicola taffensis*) in Flavobacteriia strongly correlated with the number of *A. sanguinea* cells. These bacteria did not appear after the *A. sanguinea* bloom termination. Algal extracellular products may select for specific bacterial taxa^35^, and this may explain the similarities in the specific bacterial species during the *A. sanguinea* bloom progression. HABs rely on bacteria to remineralise organic matter back to its inorganic substituents^3^.

When the *A. sanguinea* bloom was decreased (Group II) and terminated (Group III), the NH_4_^+^ and PO_4_^3−^ concentrations increased rapidly, whereas that of DOC slightly increased and then decreased. These observations were consistent with the findings of Jung et al.^15^, their outdoor mesocosm showed the increases in inorganic nutrients were caused by the release of intercellular substrates from the destruction of HABs. Cell destruction releases organic substrates that are decomposed by bacterial conversion into mineralised nutrients causing bacterial population increases^33^. In this study, the pattern changes in the total bacterial abundance time-lagged with the HABs changes, indicating that organic matter was decomposed, and that bacterial activity remained high during this stage^7^. In these stages, both Flavobacteriia and Gammaproteobacteria decreased in Group II but increased again in Group III. OTUs #02, #13 and # 09 in Flavobacteriia, and OTU #14 in Gammaproteobacteria are important common taxa in Group III. A greater number of certain bacterial species were adapted to highly eutrophic conditions. Thus, we conclude that specific bacterial diversity is influenced by bacterial lifestyles^36^. The relatively higher abundances of *Cellulophaga* (OTU #02), *Paracoccus* (#13), *Polaribacter* (#9) and *Pseudohongiella* (#14) could be attributed to their eutrophic and specific environmental adaptations. Many researchers have reported that various specific taxa of Gammaproteobacteria and Flavobacteriia are abundant at the post-HABs stage^37–39^. Therefore, the increases in certain taxa of the Gammaproteobacteria population indicate their adaptation to eutrophic conditions or the presence of eutrophic ecotypes.

Network analysis elucidates the significant associations between host–parasite, host–bacteria and bacteria–environmental factors^40,41^. For example, Needham et al.^32^ revealed the profiles and the relationship of the bacteria and virus to identify interconnectivities. In this study, the *A. sanguinea* node was associated with specific bacterial OTUs, and after the bloom, increases in specific environmental nodes (such as NH_4_^+^ and PO_4_^3−^) were associated with other bacterial OTUs. In particular, fewer bacterial–phytoplankton interactions were observed compared with those of bacteria–bacteria. This result is consistent with the finding of Cui et al.^36^. The interactions between bacteria are more complicated than those between the hosts are (here, HABs) and bacteria. Our network analysis results are consistent with those of the RDA. Thus, certain bacteria specifically respond in the HABs phycosphere, and after the termination of HABs, the changes in the environment due to the released substances from the destruction of HAB cells cause changes in other specific bacteria.

Despite this limitation, predicting the putative functional groups using FAPROTAX remains a useful alternative when metagenomic or meta-transcriptomic data are not available^42^. We revealed that the functional profiles of the groups were significantly different. During the *A. sanguinea* bloom (stage I), there was a high number of sequences assigned to chemoheterotrophy and aerobic chemoheterotrophy. These functions are categorized as broad ecosystem functions because they perform many activities and are performed by most microorganisms^43^. In particular, Amblard et al.^44^ reported that phytoplankton blooms were associated with chemoheterotrophic and photoheterotrophic metabolisms. In contrast, the proportions of chemoheterotrophy and aerobic chemoheterotrophy decreased in stages II and III, but the functional groups related to nitrogen and hydrocarbon sources were increased. These increases are consistent with the increase in inorganic nitrogen sources in the indoor microcosm. Chun et al.^45^ reported that the functional groups involved in fermentation, nitrate reduction, ureolysis, hydrocarbon degradation and aerobic ammonia oxidation were increased because of the urea introduced from the land. In addition, nitrous oxide can be produced directly by the bacterium from ammonium, nitrate and nitrite^46^. The ability to degrade and/or utilize hydrocarbon substrates is exhibited by a wide variety of bacteria. In particular, in areas containing oil spills there are metabolic increases in hydrocarbon and/or aromatic hydrocarbon degradation of specific bacterial populations^47^. Therefore, the changes in the functional groups of specific bacterial populations are consistent with the environmental impacts during and after *A. sanguinea* bloom.

Based on this result, we proposed two stages of interactions between the environmental characteristics and bacterial community in an *A. sanguinea* bloom. In the first stage termed “during *A. sanguinea* bloom”, there were marked changes in the environmental characteristics, i.e., uptake of inorganic nutrient sources (such as nitrate and phosphate) from the surrounding waters and harbouring of specific bacterial populations. In the second stage, called “late bloom / decline stage to post-bloom”, the water temperature was below 16 °C and most environmental characteristics showed dynamic changes, particularly NH_4_^+^ and PO_4_^3−^. We observed the succession in phytoplankton species from *A. sanguinea* to diatoms, particularly pennate diatoms, such as *Pseudo-nitzschia delicatissima* and *Cylindrotheca closterium*. Changes in the bacterial community were also observed. There were relatively rapid increases in Flavobacteriia and Gammaproteobacteria that remineralised the extracellular products from *A. sanguinea*. These bacteria participate in biogeochemical cycling, playing an important role in the dynamics of microbial communities. Therefore, certain bacteria taxa were adapted to more eutrophic conditions. Moreover, bacteria also change, not just the metabolic functions are consistent with the changes in environmental factors, in particular energy sources and inorganic nitrogen. Consequently, bacteria taxa and the environment dynamically change during and after *A. sanguinea* bloom. The bacterial community can rapidly respond to ecological interactions. Our research also highlighted the value of frequent sampling when evaluating bacterial community responses and interactions with phytoplankton.

## Methods

### Experimental design

The construction of the 100-L indoor microcosm has been previously described^13^ (Supplementary Fig. S2). In brief, we created three enclosures (triple replicates), each containing 100 L of natural seawater for supporting an *A. sanguinea* bloom. To observe the survival and growth of *A. sanguinea* with temperature changes, the room temperature was maintained at approximately 18 °C for the first three days and then sustained at 15 °C for 19 days. Light intensity was maintained at 50 E m^-2^ s^-1^ under a 16/8 h light/dark cycle. *A. sanguinea*-bloomed seawater was gently mixed in each microcosm using three impellers (15 cm long by 6 cm wide) at a speed of 10 rpm on an automatically controlled run/stop cycle (15 min duration each). The experiment was terminated after 22 days. We collected 17 sub-samples (daily collection for 12 d, and bi-daily collection for the last 10 d).

### Environmental parameters

The details of the environmental factors and their analysis has been previously described^10,13^. Temperature, salinity, pH and dissolved oxygen (DO) were evaluated using a portable environmental multi-parameter (YSI 6920 model, Xylem brand, OH, USA). An aliquot (100-mL) of each sub-sample was filtered through a 47-mm glass fibre filter (GF/F; Whatman, Clifton, NJ, USA). The filtered seawater was then stored in an acid-cleaned polyethylene bottle in a deep freezer at –80 °C. Subsequently, the concentrations of dissolved inorganic nutrients, such as nitrate (NO_2_^−^  + NO_3_^−^), ammonia (NH_4_^+^), phosphate (PO_4_^3−^) and silicate (SiO_2_^-^), were determined in each sample using an automatic nutrient analyser (Quaatro39; Seal Analytical Instrument, UK). To analyse the dissolved organic carbon (DOC) concentrations, an aliquot (10-mL) of each water sample was filtered through a GF/F filter (pre-combusted at 450 °C overnight) under gravity. The DOC was determined using a high-temperature catalytic combustion instrument (TOC-V_CPH_; Shimadzu, Kyoto, Japan). To determine the chlorophyll *a* concentration, each sample (500-mL) was passed through a GF/F filter under low-vacuum pressure. Each filter was soaked in cold 90% acetone/distilled water (v/v; 15-mL) and sonicated to break the cell walls. Then, chlorophyll *a* was extracted in the dark for 24 h at 4 °C. The chlorophyll *a* concentration was measured using a fluorometer (10-AU; Turner Designs, Inc., San Jose, CA, USA).

### Microscopic observation

To determine the total number of heterotrophic bacteria, an aliquot (10-mL) was collected from each sub-sample using a sterilized polyethylene bottle (15-mL). The sample was immediately fixed with glutaraldehyde at a final concentration of 2%. The sample was stored in the dark at 4 °C prior to analysis. The fixed bacterial cells were harvested through a black isopore membrane filter (GTBP 02500; Millipore, Bedford, MA, USA) and stained with 4′,6-diamidino-2-phenylinodole solution (1 μg mL^−1^)^48^. At least 600 stained bacterial cells per sample were counted using a Zeiss Axioplan epifluorescence microscope (Carl Zeiss, Oberkochen, Germany) at a magnification of 1000 × . At least 500 phytoplankton cells per sub-sample were identified and counted using a phytoplankton (or Sedgwick–Rafter) counting chamber with a light microscope (Axioplan) at a magnification of 4050 − 1000 × .

### Metagenomic next-generation sequencing of the bacterial community

For mNGS analysis, we followed the methods of Kim et al.^10^ and Jung et al.^13^. To remove the large-sized particles including planktons, samples were prefiltered using polycarbonate filters (3 μm; TSTP04700; Millipore, Bedford, MA, USA). To analyse the bacterial community, the filtrate of the 0.2–3.0 μm size fraction from the seawater (500-mL) was divided into several sections for genomic DNA (gDNA) extraction. gDNA was extracted using a DNeasy Powersoil Kit (Qiagen, Valencia, CA, USA); the DNA was diluted to a final concentration of 20 ng μL^−1^. The quantity and quality of the total gDNA were determined using a Nano-drop (Nano-MD-NS, SCINCO, Ltd., South Korea). The V3–V4 hypervariable regions of the bacterial 16S rDNA were amplified using the universal Illumina tagged forward (341F) and reverse (800R) primers (Supplementary Table S3). The amplified products from the first PCR were individually purified using a QIAquick PCR purification kit (Qiagen, Hilden, Germany). The second PCR involved 10 cycles using the tags of Nextera XT 96 index kit v2 (Illumina). DNA concentration was measured using a Bioanalyzer 2100 (Agilent Technologies, Palo Alto, CA, USA). Equal concentrations of the PCR products for each sample were pooled, merged samples were analysed using a MiSeq platform (Illumina, San Diego, CA, USA).

The resulting data were pre-processed using Mi-Seq Control Software (MCS) v2.4.1. Raw sequences analysed using FastQC^49^ for basic statistics, such as GC percentage. The quality score distribution for each base and any poor-quality sequences were identified. We also removed any ambiguous and chimeric reads, as well as the noised sequences (denoising). This procedure involved removing operational taxonomic units (OTUs) with 1, 2, and 3 reads at a cut-off rate of 97%. The processed pair-end reads were then merged using a fast length adjustment of short reads (FLASH) software tool^50^. After each sequencing procedure, a quality check was performed to remove short sequence reads (< 150 bp), low-quality sequences (score < 25 in the 16S rDNA analysis), singletons, and non-target sequences. Using the basic local alignment search tool^51^, all the sequence reads were compared with those from the National Center for Biotechnology Information database. Sequence reads with an *E*-value smaller than 0.01 were further analysed. A pairwise global alignment was performed to identify the most similar sequences. The taxonomy of the sequences with the highest similarity was assigned to the sequence read (species or genus levels with > 97% or > 94% similarity, respectively). To analyse the OTUs, CD-HIT-OTU software^52^ revealed the clustering and metagenomic functional information. To calculate the alpha-diversity, we used the closed-reference protocol published by Mothur^53^ based on the OTU table.

### Statistical analysis

Alpha diversity (including Chao1, Shannon, and Simpson diversity metrics) and heat maps were plotted through a combination of RStudio (v. 1.2.5042) using the vegan^54^, ape^55^ and ggplot2 packages^56^. Pearson’s correlation analysis examined the relationships between the measured parameters in SPSS v.12 (SAS Institute Inc., Cary, NC, USA).

The common bacterial species and environmental variables were analysed using several multivariate techniques. Before the analysis, the environmental factors data and the most abundant bacterial OTUs (each displaying a relative abundance > 1% in at least one sample) were primarily fourth root transformed to reduce skewness and to maximize the signal-to-noise ratio in the dataset^57^. Using the ranked similarity matrix, an ordination plot was produced by non-metric multidimensional scaling (nMDS) using PRIMER 6 v. 6.1.13^58^. The Bray–Curtis similarity method for hierarchical agglomerative clustering used the group average of the most abundant bacterial OTUs based on the groups selected from the nMDS analysis. To test the null hypothesis (i.e., no significant difference between the groups in the agglomerative clustering analysis), similarities were analysed with ANOSIM in the PRIMER 6 program.

A redundancy analysis (RDA) investigated the common bacterial species–environment relationships. A Pearson correlation matrix identified the correlated variables within this data set. Environmental variables below the detection limits at most sites were not included in the RDA, and only those contributing significantly to the analysis (as assessed using the forward selection) were included in the final RDA. The ratio of the eigenvalues of the constrained first axis to the second unconstrained axis was also examined because this ratio is typically indicative of an important variable in controlling common bacterial distributions^59^. The RDA was calculated with the CANOCO program version 4.55^60^. All axes were tested for significance using 999 unrestricted Monte Carlo permutations.

The correlation-based association networks are focused on the co-occurrence patterns and potential keystone taxa in bacterial interconnectivity between *A. sanguinea* and environmental factors. An extended local similarity analysis (eLSA)^61^ involved 11 datasets (bi-daily interval time of 22 days of total experimental duration) to analyse the covariation among the most abundant 19 bacterial OTUs, 3 phytoplankton groups (or species), and 10 environmental factors. *P*-values were determined using statistical approximation followed by permutation testing to reduce computing time and to ensure accuracy, whereas the *Q*-value (false discovery rate) was calculated to estimate the likelihood of false positives^62^. The eLSA network of Spearman correlation coefficients (SCC) between the variables was visualized using Cytoscape v3.7.2^63^ with *P* < 0.01 and *Q* < 0.05. The sampling was evenly spaced in two-day intervals, therefore, the maximum time-lag was 10 days. The sub-networks were selected using *A. sanguinea*, NH_4_^+^ and PO_4_^3−^ nodes and their adjacent edge types. Random undirected networks of equal sizes (based on the number of nodes and edges) were calculated using the Erdös–Rényi model in the Random Network plugin in Cytoscape.

### Functional annotation of the presented common bacterial community

A functional prediction using the functional annotation of prokaryotic taxa (FAPROTAX) and the database of metagenomics of bacterial community was used to identify ecosystem functions, although the database is not exhaustive^42^. FAPROTAX was performed using python collapse_table.py with the normalized OTU table^61^. The FAPROTAX dataset (available at http://www.zoology.ubc.ca/louca/FAPROTAX) is a manually constructed database based on cultured representatives of marine and freshwater microbiomes^45^. The functional group abundances in each module were calculated by multiplying the calculated values (“function tables”) and the total sum of the OTUs belonging to each major module.

## Supplementary Information

Supplementary Information

## Data Availability

The raw sequencing data (Fastaq files) of 16S rDNA obtained from the Mi-Seq platform were deposited in the Sequence Read Archive database at NCBI under accession numbers: SRR11282902-11,282,934 (PRJNA 611,673).
