# Supplementary material for: Dynamic bacterial community response to Akashiwo sanguinea (Dinophyceae) bloom in indoor marine microcosms
Source: Sci Rep. 2021 Mar 26;11:6983. doi: 10.1038/s41598-021-86590-8 (PMC7997919; doi:10.1038/s41598-021-86590-8)
Supplement: Supplementary file 1 — Supplementary Information [file 41598_2021_86590_MOESM1_ESM.pdf]

Supplementary information

Dynamic bacterial community response to *Akashiwo sanguinea* (Dinophyceae) bloom in indoor marine microcosms

Seung Won Jung[1,*], Junsu Kang[1,2], Joon Sang Park[1], Hyoung Min Joo[3], Sung-Suk Suh[4], Donhyug Kang[5], Taek-Kyun Lee[6], Hyun-Jung Kim[1]

[1]Library of Marine Samples, Korea Institute of Ocean Science & Technology, Geoje, 53201, [2]Department of Oceanography, Pukyoung National University, Busan 48513, Republic of Korea, [3]Division of Polar Ocean Science, Korea Polar Research Institute, Incheon 21990, Republic of Korea, [4]Department of Bioscience, Mokpo National University, Muan 58554, Republic of Korea, [5]Maritime Security Research Center, Korea Institute of Ocean Science & Technology, Busan 49111, Republic of Korea, [6]Risk Assessment Research Center, Korea Institute of Ocean Science & Technology, Geoje 53201, Republic of Korea

***Correspondence to:** S.-W. Jung, diatoms@kiost.ac.kr, Fax: +82-55-639-8429

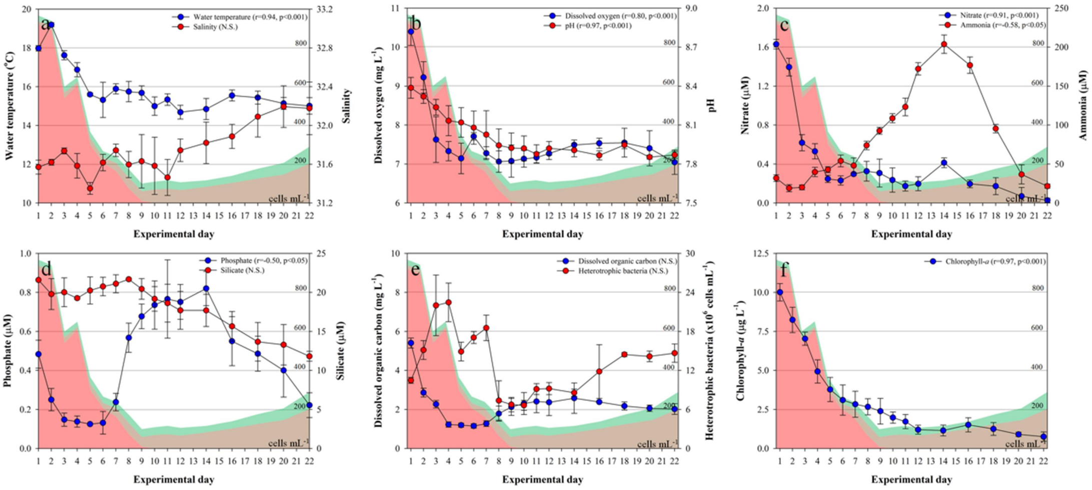

Supplementary Figure S1. Changes in the environmental characteristics of the indoor microcosm bloom dynamics of *Akashiwo sanguinea*. (a) Water temperature and salinity, (b) dissolved oxygen and pH, (c) nitrate (nitrate + nitrite) and ammonia, (d) phosphate and silicate, (e) dissolved organic carbon and heterotrophic bacteria and (f) chlorophyll *a*. The *r* value in each figure (upper right) is the Pearson correlation coefficient (n = 17). Data are presented as the mean (three replicates) and error bars indicate the standard deviation. N.S. indicates no significance.

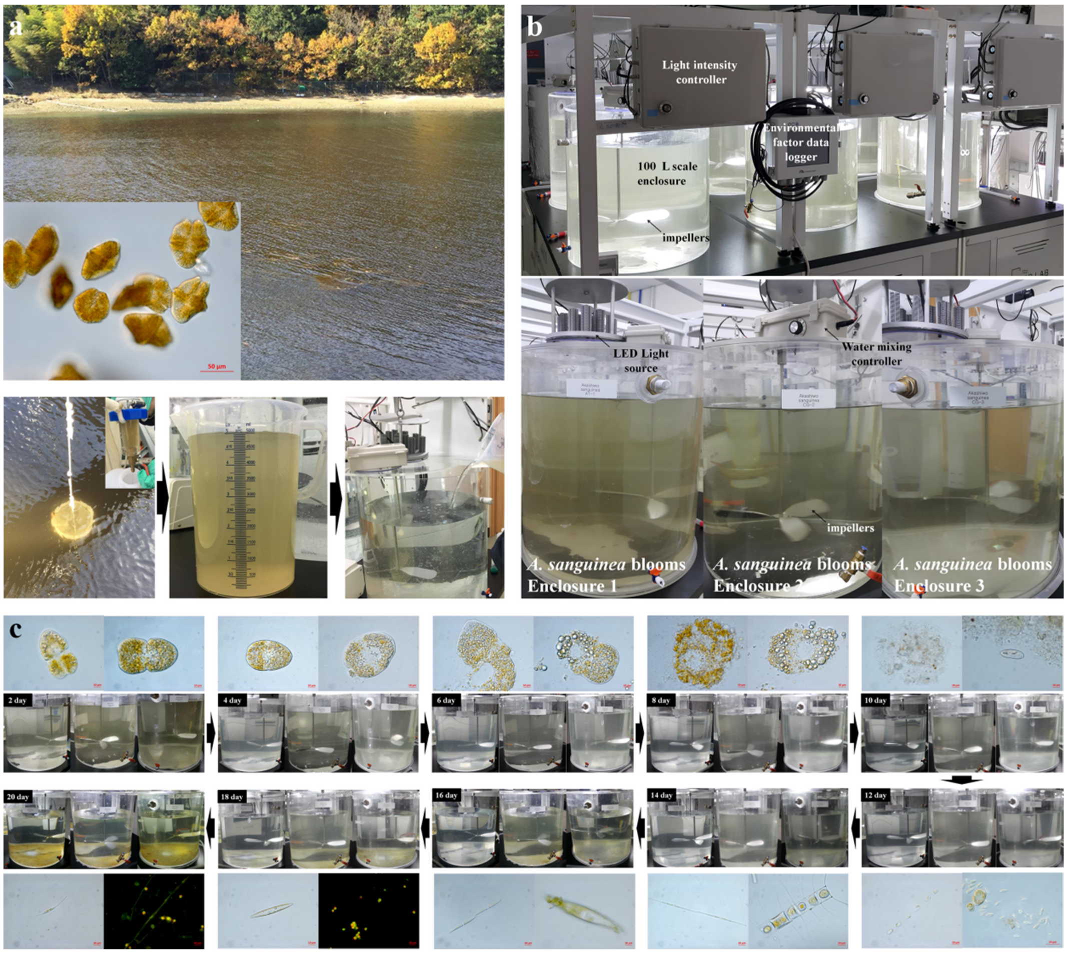

Supplementary Figure S2. Experimental design of the 100-L indoor microcosm bloom dynamics of *Akashiwo sanguinea*. (a) *A. sanguinea* bloom in the Jangmok Bay time-series monitoring site (JBTMS: 34°59′37′′ N and 128°40′27′′ E) and microcosm using concentrated *A. sanguinea* cells in natural seawater. (b) Triplicate indoor microcosm experiments in seawater. Each enclosure consisted of a LED light with light intensity controller, impellers with water mixing controller and data logger of environmental factors. (c) Time-series photographs of the three replicate microcosms and microscopic photographs of common phytoplankton.

Supplementary Table S1. Abbreviations and node sizes of the significant factors (*P*<0.01; *Q*<0.05) identified in the Network Analysis provided in Figure 4.

Factor	Abbreviation	Node size	Classification
Water temperature	WT	1.99	Environmental factor
Salinity	Sal.	2.37	
pH	pH	1.68	
Dissolved oxygen	DO	1.66	
Nitrate (including Nitrite)	NO3	0.71	
Ammonia	NH4	2.93	
Phosphate	PO4	0.80	
Silicate	SiO2	2.03	
Dissolved organic carbon	DOC	1.19	
Chlorophyll-*a*	DO	1.19	
Akashiwo sanguinea	*A.san.*	1.56	Autotroph
Diatoms	Diatoms	2.88	
Other phytoplankton	Oth.Phyto.	2.53	
Total bacteria	Bacteria	1.87	Bacteria
Marinovum algicola	OTU #00	2.34	
Magnetospira thiophila	OTU #01	1.47	
Cellulophaga tyrosinoxydans	OTU #02	1.15	
Polaribacter marinivivus	OTU #03	0.79	
Marivita roseacus	OTU #04	1.06	
Methylotenera versatilis	OTU #05	1.01	
Tenacibaculum aestuariivivum	OTU #06	1.42	
Halobacteriovorax marinus	OTU #07.	0.77	
Fluviicola taffensis	OTU #08	0.60	
Polaribacter atrinae	OTU #09	1.10	
Polaribacter huanghezhanensis	OTU #10	0.78	
Owenweeksia hongkongensis	OTU #11	0.68	
Tenacibaculum aiptasiae	OTU #12	1.20	
Paracoccus mangrovi	OTU #13	0.94	
Pseudohongiella spirulinae	OTU #14	0.82	
Polaribacter pacificus	OTU #15	0.84	
Nereida ignava	OTU #18	0.38	
Thalassobacter stenotrophicus	OTU #20	0.51	

Supplementary Table S2. Local similarity correlations of sub-networks I-III in the Network Analysis in Figure 4.

Sub-network	X	Y	LS	Xs	Ys	Length	Delay	SCC	P	Q
I	*A.san.*	OTU #01	-0.592	1	1	11	0	-0.964	<0.001	<0.001
	A.san	OTU #04	-0.963	1	1	11	0	-0.853	0.009	0.010
	A.san	OTU #12	-0.963	1	4	8	-3	0.189	0.009	0.010
	A.san	Diatoms	-0.963	1	1	11	0	-0.853	0.005	0.008
	pH	*A.san.*	1.240	1	1	11	0	0.823	0.005	0.008
	Chl-*a*	*A.san.*	0.998	1	1	11	0	0.863	0.002	0.005
II	PO_4^{3-}	Bacteria	-0.539	1	1	11	0	-0.900	0.007	0.010
	PO_4^{3-}	OTU #05	0.579	1	1	11	0	0.909	<0.001	<0.001
	PO_4^{3-}	OTU #11	0.562	1	1	11	0	0.909	0.001	0.003
II	NH_4^+	OTU #07	0.549	1	2	9	-1	0.484	0.006	0.009
	NH_4^+	OTU #10	-0.568	1	2	10	-1	-0.770	0.001	0.003
	NH_4^+	OTU #11	0.567	1	1	11	0	0.882	<0.001	<0.001
III	Diatoms	Oth.Phyto.	0.547	1	1	11	0	0.808	0.001	0.003
	Diatoms	OTU #04	0.623	1	1	11	0	0.982	<0.001	<0.001
	Diatoms	OTU #07	0.535	1	1	11	0	0.813	0.009	0.011
	Diatoms	OTU #08	-0.571	1	1	11	0	-0.936	<0.001	<0.001
	Diatoms	OTU #13	0.607	1	1	11	0	0.973	<0.001	<0.001
	Diatoms	OTU #14	0.523	2	2	10	0	0.800	0.001	0.003
	Sal	Diatoms	0.551	1	1	11	0	0.843	0.010	0.011
	pH	Diatoms	-0.542	1	1	11	0	-0.815	0.004	0.008
	SiO_2^-	Diatoms	-0.548	1	1	11	0	-0.900	0.001	0.003
	NO_3^-	Diatoms	-0.560	1	1	11	0	-0.855	0.001	0.003
	Chl-*a*	Diatoms	-0.578	1	1	11	0	-0.891	<0.001	<0.001
	A.san	Diatoms	-0.963	1	1	11	0	-0.853	0.005	0.008

Abbreviations: LS, local similarity; Xs and Ys, the initial day of the LS correlation; Length, the duration of the LS correlation in days; Delay, time-lag in days; SCC, Spearman's Correlation Coefficient; P and Q, P-value and Q-value, respectively.

Supplementary Table S3. Primers used in the PCR amplification of V3–V4 regions in the 16S rDNA analysis.

Target region	Step	Primer		PCR reaction mixtures (25-µL)	PCR condition	References
		Forward	Reverse			
16S rDNA (V3-V4 region)	First PCR	Ilumina preadapter +Sequencing primer sequence+341F (5'-CCTACGGGN GGCWGCAG-3′)	Ilumina preadapter +Sequencing primer sequence+800R (5'-TACCAGGGT ATCTAATCC-3′)	200 µmol L^{-1} each dNTP, 1.5 mmol L^{-1} $MgCl_2$, 0.3 µmol L^{-1} each primer, 2.5 U Taq DNA polymerase (TaKaRa, EX Taq, Kyoto, Japan), and DNA template (20 ng μL^{-1})	initial denaturation step at 95 °C for 3 min, followed by 30 cycles of denaturation at 95 °C for 10 s, annealing at 52 °C for 45 s, and extension at 72 °C for 1 min, and a final extension step of 72 °C for 5 min.	1, 2
	Second PCR	Ilumina preadapter +Sequencing primer sequence+341F (5'-CCTACGGGN GGCWGCAG-3')	Ilumina preadapter +Sequencing primer sequence+800R (5'-TACCAGGGT ATCTAATCC-3′)	200 µmol L^{-1} each dNTP, 1.5 mmol L^{-1} $MgCl_2$, 0.3 µmol L^{-1} each primer, 2.5 U Taq DNA polymerase (TaKaRa, EX Taq, Kyoto, Japan), and DNA template (800 ng), and Nextera XT index Kit (Illumina, CA, USA)	initial denaturation step at 95 °C for 3 min, followed by 12 cycles of denaturation at 95 °C for 10 s, annealing at 52 °C for 45 s, and extension at 72 °C for 1 min, and a final extension step of 72 °C for 5 min.	
	Pooling	Triplicate reaction products (with a consistent volume) were pooled and purified using an Qiaquick PCR purification Kit (No. 28104, Qiagen Inc.). Their DNA concentration was measured in a Bio-analyzer 2100 (Agilent Technologies, Palo Alto, CA, USA).				

References

1. Herlemann, D. P. R. *et al.* Transitions in bacterial 80 communities along the 2000 km salinity gradient of the Baltic Sea. *The ISME Journal* **5**, 1571-1579 (2011).

2. Ogihara, H. *et al*. Microfloral and chemical changes of salted pickles (Suguki) during its manufacturing process. *Japanese Journal of Food Microbiology* **26**, 98-106 (2009).
